# Analysis of crotonaldehyde puff-by-puff release in mainstream cigarette smoke under various smoking regimens by high-performance liquid chromatography with the modified QuEChERS method

**DOI:** 10.1098/rsos.172003

**Published:** 2018-06-13

**Authors:** Chao Li, E'xian Li, Jian Zhang, Ying Tao, Chengming Zhang, Xiaofeng Shen, Ya Liu, Yunhua Qin

**Affiliations:** 1 Technology Center of Yunnan Tobacco Industry Co., Ltd., Yunnan, People's Republic of China; 2 Yunnan Academy of Agricultural Sciences, Institute of Biotechnology and Genetic Resources, Yunnan, People's Republic of China

**Keywords:** RM20H smoking machine, QuEChERS, intensive smoking regimens, crotonaldehyde, puff-by-puff release

## Abstract

To study puff-by-puff release characteristics of crotonaldehyde in mainstream cigarette smoke under diverse intensive smoking regimens, we designed an RM20H smoking machine with a puff-by-puff smoke collection unit to automatically trap crotonaldehyde in the mainstream cigarette smoke. Using this process, we trapped, puff-by-puff, crotonaldehyde in mainstream smoke generated by different smoking regimens and quantitatively analysed the levels of crotonaldehyde using high-performance liquid chromatography with a modified QuEChERS sample pretreatment method. On the basis of the crotonaldehyde in each puff, we determined crotonaldehyde's puff-by-puff release characteristics. The results showed that crotonaldehyde's puff-by-puff release remained nearly constant for the International Organization for Standardization mode while increased polynomial trend was seen (*n* ≥ 6) under the Massachusetts and Health Canada smoking regimens. The equation fit for various regimens was good (*R*^2^ > 0.9192). Release characteristics by puff were classified into four categories: (1) first, second and third puffs; (2) fourth and fifth puffs; (3) sixth puff; and (4) seventh and eighth puffs.

## 1. Introduction

Cigarette smoking machines are used to simulate consumer smoking behaviour, which is important for the study of the chemical composition of mainstream cigarette smoke. Many government regulatory bodies have developed relevant policies and regulations on various parameters of smoking machines according to the rules set by the International Organization for Standardization (ISO) Technical Committee (specifically for tobacco, under the subcommittee of ISO/TC 126) [1]. The ISO standard regimen for machine smoking, like other standard machine regimens, cannot fully represent average human smoking behaviour [2]. As debate on the relevance of the ISO smoking parameters continues in the literature, it is important to study the effect of smoking regimens on chemical release because it affects the combustion of cigarettes [3]. Therefore, study of the effects of different smoking regimens (including the Massachusetts and Health Canada intensive smoking regimens) on puff-by-puff chemical composition in cigarette mainstream smoke has an increasingly important role in the accurate analysis of smoke emission [4]. Currently, in China, much of the research on smoke tar, nicotine, water, ammonia, carbon monoxide, the main compounds of phenols, tobacco-specific nitrosamines, hydrogen cyanide and nitrogen oxides emissions is being conducted under different cigarette smoking regimens [3,5–12]. However, few reports have described crotonaldehyde's release and the factors that may influence its levels in mainstream smoke. For example, a study by Xie *et al.* [13]. on cigarette fire temperature distribution described the seven harmful components in mainstream smoke. Li and co-workers [14] studied the release of six chemical constituents by puff in ISO mode using a transformation smoking machine. Chen [15] studied the relationship between important parameters and the release of crotonaldehyde in mainstream smoke by changing key process parameters in the method.

Many methods used to analyse the release of crotonaldehyde in mainstream smoke have been reported, some of which have used high-performance liquid chromatography (HPLC) for quantitative analysis of a whole cigarette [16,17]. Puff-by-puff release analysis of crotonaldehyde in mainstream smoke has not been reported. In particular, when carrying out an extraction process from a Cambridge filter, filter fines are usually suspended in the filtrate, leading to filtration difficulties and severe matrix effects. Therefore, it is necessary to develop a simple, effective and reliable method for quantitative analysis of the puff-by-puff release of crotonaldehyde.

Our study presents a simple HPLC with modified QuEChERS sample pretreatment method for the effective quantification of the puff-by-puff release of crotonaldehyde in mainstream cigarette smoke, under different smoking regimens. Additionally, we used several statistical methods to provide a reference for reducing crotonaldehyde levels in mainstream cigarette smoke.

## 2. Experiment

### 2.1. Materials and reagents

#### 2.1.1. Specification of cigarette samples

We coded four types of domestic commercial cigarettes as A, B, C and D. Cigarettes B and C were high-grade tobacco with a high rate of total sugar and reducing sugar; cigarettes A and D were low-grade tobacco with a low rate of total sugar and reducing sugar. All four types of cigarettes were 84 mm in length and had a circumference of 24.3 mm; other parameters are given in table 1.

**Table 1.** Specifications of cigarette samples.

cigarette sample	product price class	cigarette length (mm)	filter length (mm)	tar (mg/cigarette)	nicotine (mg/cigarette)	CO (mg/cigarette)	total ventilation level (%)
A	third class	59	25	11	1.1	12	25 ± 10
B	first class	59	25	11	1.1	12	25 ± 10
C	first class	54	30	8	0.8	10	22 ± 10
D	third class	59	25	10	1.0	11	32 ± 10

#### 2.1.2. Reagents and instruments

We purchased acetonitrile, tetrahydrofuran and isopropanol (HPLC grade) from Merck (Darmstadt, Germany). Perchloric acid (≥36% purity) and pyridine (HPLC grade) were purchased from Nanjing Chemical Reagent Company Inc. (Nanjing, China). 2,4-Dinitrophenyl-hydrazine (DNPH), formaldehyde, aldehyde, acetone, acrolein, propanal, crotonaldehyde, 2-butanone and butyl aldehyde 2,4-dinitrophenylhydrazone derivative compounds (≥97% purity) were purchased from Acros (Geel, Belgium). Anhydrous magnesium sulfate (MgSO_4_; HPLC grade) and sodium chloride (HPLC grade) were purchased from Kermel Company Inc. (Tianjin, China). C18, primary secondary amine and graphitized carbon black (GCB) (all 40–60 µm) were obtained from Bonna-Agela Technologies (Tianjin, China). Ferric chloride (HPLC grade) and ferrous chloride (HPLC grade) were purchased from Damao Chemical Reagent Factory (Tianjin, China). Ultrapure water was prepared from a Milli-Q Reagent system (Millipore, Billerica, USA) and was consistent with the GB/T 6682 requested level. Other reagents were of analytical grade.

We used an RM20H smoking machine (Borgwaldt KC, Germany), an AT201 electronic balancer (Mettler-Toledo, Switzerland), a Waters 2695 HPLC system (Waters, Milford, MA, USA), an 8510 ultrasonic generator (Branson, St Louis, MO, USA), a QL-866 vortex mixer (Qilinbeier, China) and a TGL-16M high-speed desktop refrigerated centrifuge (Hunan Instrument, China).

### 2.2. Parameters of ISO, Massachusetts and Health Canada smoking regimens

We used three different smoking modes to study the release of crotonaldehyde. Differences in the puffing parameters among the ISO, Massachusetts and Health Canada smoking regimens included puffing volume, puffing frequency and puffing rate. They all had the same puffing duration of 2.0 s as listed in table 2.

**Table 2.** Different parameters of intensive smoking regimens.

smoking mode	puffing volume (ml)	puffing frequency (s)	puffing duration (s)	filter ventilation blocking (%)
ISO	35 ± 0.5	60 ± 0.5	2 ± 0.5	0
Health Canada	55 ± 0.5	30 ± 0.5	2 ± 0.5	100
Massachusetts	45 ± 0.5	30 ± 0.5	2 ± 0.5	50

### 2.3. Preparation of standard solutions

Formaldehyde (50 mg), acetaldehyde (100 mg), acetone (50 mg), acrolein (40 mg), propionaldehyde (30 mg), crotonaldehyde (30 mg), and 2-butanone (30 mg), butyl aldehyde (30 mg) 2,4-dinitrophenylhydrazone derivative compounds were accurately weighed and dissolved in 30 ml acetonitrile in 50 ml beakers to produce a stock standard solution. Primary calibration solutions were prepared by diluting 10 ml stock standard solutions in 50 ml volumetric flasks with acetonitrile.

Concentrations used for constructing the calibration curve were 0.2, 0.4, 1, 2, 10 and 50 µg ml^−1^. Then, working solutions were prepared by transferring primary calibration solutions (0.1, 0.2, 0.5, 1.5 and 25 ml) into 100 ml acetonitrile again, respectively. Series of standard solution of six different concentrations of crotonaldehyde (0.2, 0.4, 1, 2, 10 and 50 µg ml^−1^) were prepared for formaldehyde, acetaldehyde, acetone, acrolein, propionaldehyde, crotonaldehyde, and 2-butanone, butyl aldehyde 2,4-dinitrophenylhydrazone derivative compounds, which were treated as standard calibration solutions. The calibration solution of the carbonyl group is prepared only before usage.

### 2.4. Sample preparation

We followed the requirements of GB/T 19609-2004 [18] for connecting to the RM20H puff-by-puff unit. Twenty cigarettes were included for the traps of total particulate matter from cigarette smoke under the ISO model while 10 cigarettes were included for the Massachusetts and Health Canada smoking modes. We measured each cigarette sample twice and trapped the total particulate matter on Cambridge filters. After the cigarette-smoking using the RM20H puff-by-puff unit, two blank puffs are required. Then the trapper was taken out and left to stand for 3 min. We added DNPH to the filter to react with all carbonyl compounds. A glass fibre filter was used to trap the total particulate matter puff-by-puff in the mainstream smoke. After the process was completed, we placed the filter into a 100 ml Erlenmeyer flask and added 50 ml of pyridylacetonitrile. The solution was ultrasonicated for 10 min at 30°C. Then, we immediately vibrated the solution for l min after adding 3 g of sodium chloride and 12 g of anhydrous MgSO_4_. We centrifuged the obtained solution for 5 min and separated 1 ml supernatant. After centrifugation, we simultaneously added 100 mg of anhydrous MgSO_4_, 10 mg GCB, 10 mg C_18_, and 40 mg iron oxide (Fe_3_O_4_) magnetic nanoparticles [19] into the supernatant. With a 30 s vortex, we collected the supernatants using an applied magnetic field. Treated analytes appeared to be clear and transparent, without any filter impurities; 2 ml supernatant was transferred to a chromatographic flask and we detected the concentration of crotonaldehyde using the HPLC method.

### 2.5. Chromatographic condition

We performed this analysis using a ZORBAX Eclipse XDB-C18 column (50 mm × 4.6 mm, 1.8 µm particle size, Agilent, Santa Clara, CA, USA). Optimizing chromatographic operating conditions were based on the requirement of the resolution between the respective peaks. Conditions were as follows. Mobile phase A: water : acetonitrile : tetrahydrofuran : isopropanol (59 : 30 : 10 : 1); mobile phase B: water : acetonitrile (35 : 65); column temperature: 30°C; column flow: 0.8 ml min^−1^; volume injection: 5 µl; gradient: 0 min: mobile phase A 100%, mobile phase B 0%, 20 min: mobile phase A 60%, mobile phase B 40%; detector: UV detector; and time analysis: 40 min.

We identified the carbonyl compounds using HPLC. We obtained eight points of carbonyl compounds in the peak area. Using these peak areas as the ordinate, we set concentrations of carbonyl compounds as the abscissa. We set eight kinds of carbonyl compounds by a calibration curve. For the linear regression calibration data, we determined that for each compound R^2^ should be not less than 0.990. After determination of the extracted sample, we calculated the eight concentrations of the sample carbonyl compounds according to the area of the fingerprint spectrum of these compounds extracted from the smoke sample of every cigarette.

### 2.6. Data analysis

ChemPattern software (Chemmind Technologies Co., Beijing, China) was used to analyse crotonaldehyde release data in mainstream smoke on a puff-by-puff basis. We used descriptive statistics, scatter trends and multiple correlation analysis to evaluate release trends on a puff-by-puff basis.

## 3. Method validation

### 3.1. Recovery of modified QuEChERS

The validity of the modified QuEChERS method was estimated by means of recovery experiments and all experiments were conducted in duplicate. The average recovery of the target compound ranged from 91.5% to 110.7%, and the relative standard deviation was not more than 5.5%. The recoveries and relative standard deviations were in line with crotonaldehyde analysis requirements.

### 3.2. Chromatographic behaviour

**Figure 1.**
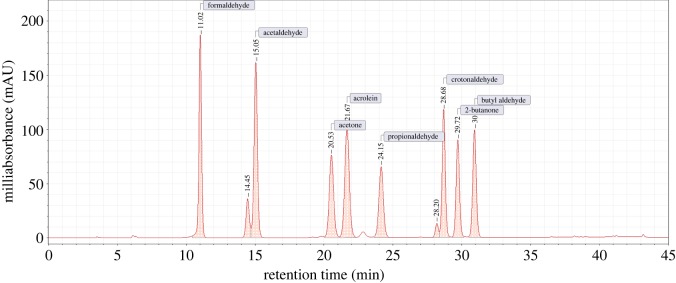
Chromatogram of standard solution.

**Figure 2.**
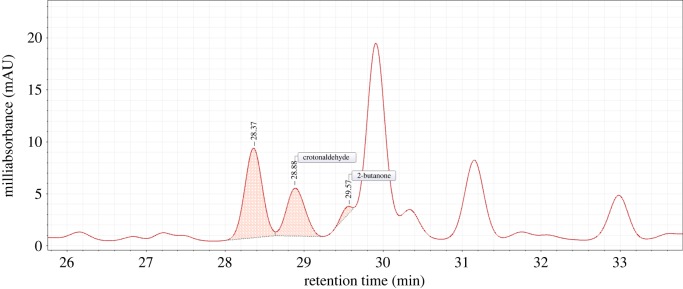
Chromatogram of sample.

We obtained test results of crotonaldehyde release on a puff-by-puff basis from the cigarette samples and analysed the results under different intense smoking regimens. The retention time of crotonaldehyde was 28.88 min. Standard and sample chromatograms are shown in figure 1 and figure 2, respectively. Furthermore, the resolution, theory plate and symmetry factor of peaks are shown in table 3.

**Table 3.** Parameters of chromatographic peaks.

compound name	resolution	theory plate	symmetry factor	retention time
formaldehyde	n/a	17 057	0.99	11.02
acetaldehyde	1.35	18 549	0.92	15.05
acetone	10.66	19 621	1.06	20.53
acrolein	1.88	19 632	1.05	21.67
propionaldehyde	4.10	26 478	1.00	24.15
crotonaldehyde	1.14	75 084	1.03	28.68
2-butanone	2.37	69 156	1.03	29.72
butyl aldehyde	2.63	68 661	1.00	30.93

### 3.3. The limit of detection and limit of quantitation

We used the following experimental design and calculations. (1) We used acetonitrile as the solvent blank and performed 20 consecutive tests using a chromatograph. The standard deviation (s_b1_) of the blank was calculated, as shown in table 4. (2) We calculated the value of *k* (a constant related to confidence). (3) We formulated the crotonaldehyde standard series solution in the concentration range 0.2, 0.4, 1, 2, 10 and 50 µg ml^−1^, and calculated sensitivity (*s*) at low concentrations based on the response signal. (4) We calculated the detection limit of the method according to equation (3.1):

3.1 $$q_{L}=\frac{ks_{b1}}{s},$$

where *q*_L_ is the minimum detectable concentration, *k* is a constant related to confidence, *s_b1_* is the standard deviation value for the blank, and *s* is the sensitivity at low concentrations, which is the slope of the standard curve in the low concentration range (table 4).

**Table 4.** Twenty consecutive test data using blank.

injection number	concentration (μg ml^−1^)	injection number	concentration (μg ml^−1^)
1	0.194	11	0.187
2	0.203	12	0.215
3	0.185	13	0.198
4	0.171	14	0.176
5	0.202	15	0.205
6	0.203	16	0.188
7	0.204	17	0.169
8	0.175	18	0.177
9	0.183	19	0.193
10	0.171	20	0.210
mean value (μg ml^−1^)		0.1905	
standard deviation		0.0142	

Setting *k* at 3, we obtained the slope of the standard curve as *s* = 0.936. Therefore, the detection limit *q*_L_ = 3 × 0.0142/0.936 = 0.0455 µg ml^−1^. According to the test's dilution and concentration conditions, we obtained the detection limit (per puff) of this method as *q_L_'* = 0.0455 × 50/20 = 0.1138 µg/cigarette. The limit of quantification *q*_L_′′ = 0.1138 × 10/3 = 0.379 µg/cigarette was far lower than the crotonaldehyde content of a minimum 1.410 µg analysed per puff in a cigarette. The relative standard deviation of analysed = s.d./mean × 100% = 0.0142/0.1905 × 100% = 7.45%. Therefore, the method can be used to analyse levels of crotonaldehyde in ordinary cigarettes on a puff-by-puff basis.

## 4. Results

### 4.1. Descriptive statistics of crotonaldehyde per puff in mainstream smoke under ISO regime

Descriptive statistics of the samples are listed in table 5. As shown in the table, there was a range of crotonaldehyde release between the different samples. The overall release ranges from 1.41 to 1.72 µg/puff. Crotonaldehyde release per puff of each sample had relatively small changes among different samples. We observed the following mean values of release in different samples: the release in the A samples was the highest (1.673 µg/cigarette), and the release in the D samples was the lowest (1.470 µg/cigarette). In addition, from the variance data it can be observed that different samples of crotonaldehyde release had a distinctive trend, that is, the maximum and minimum variances were 0.060 and 0.024, respectively.

**Table 5.** Descriptive statistics of crotonaldehyde release by puff in different cigarette samples.

sample	minimum	maximum	mean value	s.d.	variation coefficient
A	1.64	1.72	1.673	0.027	0.0007
B	1.59	1.66	1.633	0.024	0.0006
C	1.43	1.57	1.487	0.044	0.0020
D	1.41	1.58	1.470	0.060	0.0036

### 4.2. Poincaré scatter trend simulation line plot analysis

**Figure 3.**
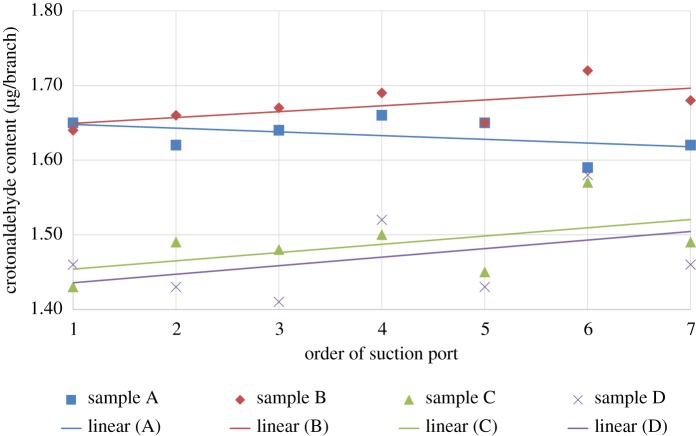
Poincaré scatter trend simulation line plot of crotonaldehyde release by puff for different samples in ISO mode.

Figure 3 describes the Poincaré scatter trend simulation line plot of the different samples under the ISO mode. The figure shows that the release of crotonaldehyde in the same sample remained nearly steady during the puff-by-puff smoking process, but this release varied among the different samples. The largest release by puff came from sample A. The trend equations for the different samples were as follows: *y_A_ = *1.6729; *y_B_ = *1.6329; *y_C_ = *1.4871; and *y_D_ = *1.4700. As a result, this study showed that levels of crotonaldehyde released by puff remained linear with a slope of almost 0.

## 5. Discussion

### 5.1. Comparison of crotonaldehyde release in mainstream smoke among ISO, Massachusetts and Health Canada intensive regimens

**Figure 4.**
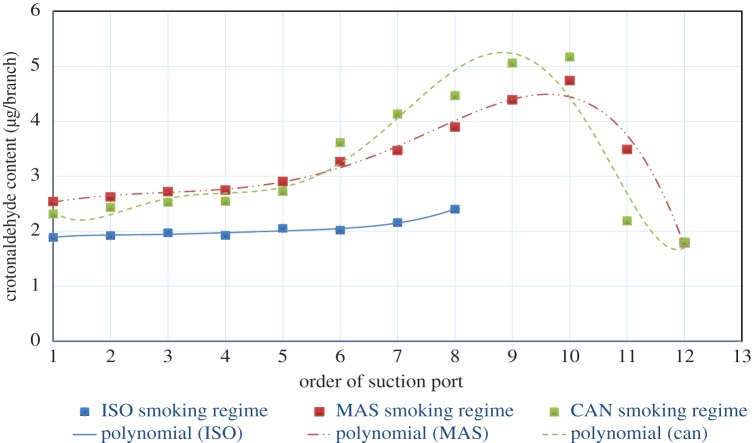
Poincaré scatter trend simulation line plot of crotonaldehyde release by puff for different intensive regimens.

Figure 4 shows the Poincaré scatter trend simulation line plot for crotonaldehyde release. The trend line equations are as follows: *y*_ISO_ = −5E-05*x*^6^ + 0.0016*x*^5^ − 0.0194*x*^4^ + 0.1136*x*^3^ − 0.342*x*^2^ + 0.5174*x* + 1.6175, *R*² = 0.9682; *y*_MAS_ = 0.0003*x*^6^ − 0.0125*x*^5^ + 0.17*x*^4^ − 1.1006*x*^3^ + 3.5488*x*^2^ − 5.1861*x* + 4.9417, *R*² = 0.9192; and *y*_CAN_ = −0.0002*x*^5^ + 0.0032*x*^4^ − 0.0076*x*^3^ − 0.0478*x*^2^ + 0.2765*x* + 2.3045, *R*² = 0.9754. The figure shows that some similarities emerge relative to crotonaldehyde release under the different intensive puffing modes. In the ISO mode, crotonaldehyde release by puff demonstrates a polynomial trend increase (*n* ≥ 6), the *R*^2^ = 0.9682, and the proposed combined degree of curve fitting was general. In Massachusetts mode, crotonaldehyde release by puff demonstrates a polynomial trend and the release gradually decreased after the ninth puff, the *R*^2^ = 0.9192 and the degree of curve fitting was ordinary. In the Health Canada mode, crotonaldehyde release by puff also showed a polynomial trend, the release gradually decreased after the ninth puff, the *R*^2^ = 0.9754 and the degree of curve fitting was relatively better.

### 5.2. R-clustering analysis of crotonaldehyde release by puff for different intensive regimens

**Figure 5.**
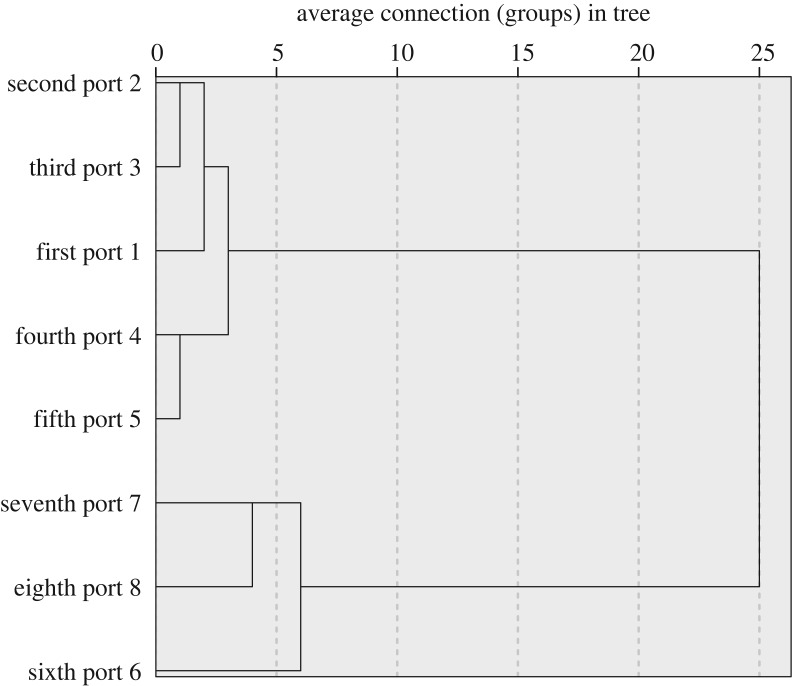
Crotonaldehyde release by puff: R-clustering hierarchical graph by different suction mode.

We used the R-clustering analysis method to study the release characteristics of crotonaldehyde by puff for different intensive regimens (links between cluster groups, using Euclidean distance). From the cluster genealogy chart (figure 5), the first eight puff features can be divided into four categories according to the different intensive regimens: (1) the first, second and third puffs; (2) the fourth and fifth puffs; (3) the sixth puff; and (4) the seventh and eighth puffs.

## 6. Conclusion

We used an RM20H smoking machine with an automated puff-by-puff trapping unit to collect crotonaldehyde from total particulate matter on a Cambridge filter for three different smoking regimens. We conducted a quantitative analysis using an HPLC external standard technique with a modified QuEChERS sample pretreatment method. We observed the treated analytes to be clear and transparent, without any filter impurities. The retention time of the crotonaldehyde was 28.907 min. The limit of detection (0.1138 µg/cigarette), the limit of quantification (0.379 µg/cigarette) and the RSD (7.45%) all met the detection needs for puff-by-puff release analysis. In ISO mode, the release of crotonaldehyde for the same sample remained almost the same during the puff-by-puff smoking process, but the release varied among the different samples within a range of 1.41–1.72 µg per cigarette. The largest release by puff was from sample A (1.673 µg per cigarette). In the Massachusetts and Health Canada modes, the crotonaldehyde release demonstrated a polynomial trend, and this release gradually decreased after the ninth puff (*R*^2^ ≥ 0.919). The R-cluster analysis showed that the first eight puff features could be divided into four categories according to the different intensive regimens: (1) first, second and third puffs; (2) fourth and fifth puffs; (3) sixth puff; and (4) seventh and eight puffs.

## Data accessibility

This article does not contain any additional data.
